# Disappearance of Seasonal Respiratory Viruses in Children Under Two Years Old During COVID-19 Pandemic: A Monocentric Retrospective Study in Milan, Italy

**DOI:** 10.3389/fped.2021.721005

**Published:** 2021-08-05

**Authors:** Giulio Ippolito, Adriano La Vecchia, Giulia Umbrello, Giada Di Pietro, Patrizia Bono, Stefano Scalia Catenacci, Raffaella Pinzani, Claudia Tagliabue, Samantha Bosis, Carlo Agostoni, Paola Giovanna Marchisio

**Affiliations:** ^1^University of Milan, Milan, Italy; ^2^Fondazione IRCCS Ca' Granda Ospedale Maggiore Policlinico, Laboratory of Virology, Milan, Italy; ^3^Fondazione IRCCS Ca' Granda Ospedale Maggiore Policlinico, Pediatric Intensive Care Unit, Milan, Italy; ^4^Fondazione IRCCS Ca' Granda Ospedale Maggiore Policlinico, Pediatric Highly Intensive Care Unit, Milan, Italy; ^5^Fondazione IRCCS Ca' Granda Ospedale Maggiore Policlinico, Pediatric Intermediate Care Unit, Milan, Italy; ^6^Department of Clinical Sciences and Community Health (DISCCO), University of Milan, Milan, Italy; ^7^Department of Pathophysiology and Transplantation, University of Milan, Milan, Italy

**Keywords:** respiratory viruses, children, COVID-19, epidemiology, public health

## Abstract

**Background:** The containment measures adopted during COVID-19 pandemic have influenced the epidemiology of other respiratory viruses.

**Aim:** We analyzed the modification of the incidence and etiology of lower respiratory tract infections (LRTIs) in young children during COVID-19 pandemic.

**Methods:** Case series of all children under 2 years old hospitalized at a tertiary care Hospital in the Center of Milan, Italy diagnosed with LRTIs in three consecutive winter seasons (from the 1st of November to the last day of February in 2018/2019, 2019/2020 and 2020/2021). We compared the number of hospitalizations and viral detections in the 2020/2021 with the average of 2018/2019 and 2019/2020 (pre-COVID-19) using the Poisson distribution.

**Results:** we enrolled 178 patients (66 from 2018/2019, 96 from 2019/2020, 16 from 2020/2021) 94 males (53%) and 84 females (47%), with a median (IQR) age of 5 (2–13) months. The number of hospitalizations during the 2020/2021 season was 80% lower than the average of the pre-COVID-19 seasons (16 vs. 81, *p*<*0*.001). Overall, 171 (96%) patient's nasopharyngeal aspirate (NPA) detected at least one virus (110, 64%, single-detection, 61, 36%, co-detections). In 2020/2021 we observed the disappearance of Respiratory Syncytial virus (0 vs. 54, *p* < 0.001), Influenza virus (0 vs. 6.5, *p* = 0.002), Metapneumovirus (0 vs. 8, *p* < 0.001), Parainfluenza viruses (0 vs. 3.5, *p* = 0.03) and a significant reduction of Adenovirus (2 vs. 7, *p* = 0.03), Bocavirus (2 vs. 7.5, *p* = 0.02) and Enterovirus (1 vs. 5, *p* = 0.04). No significant difference was found for Rhinoviruses (14 cases vs. 17, *p* = 0.2), other Coronaviruses (0 vs. 2, *p* = 0.1), and Cytomegalovirus (1 vs. 1, *p* = 0.7).

**Conclusions:** We observed a striking reduction in hospitalizations due to LRTIs and a modification of the etiology, with enveloped viruses mainly affected.

## Introduction

COVID-19 pandemic is the major medical emergency of the 21st century for both healthcare professionals and the general population. For a disease lacking specific therapy, prevention is mandatory to reduce its morbidity and lethality rate. Vaccination is a very promising tool for future prevention (1–5). Before the achievement of a wide spread vaccination coverage, non-pharmacological interventions (NPI) have been and continue to be widely recommended by most Governments to contain the spread of SARS-CoV-2. Using facial masks, social distancing, adequate hand hygiene, surface disinfection and ventilation of indoor spaces are recommended by National Governments and extensively used by the general population (6). Despite the severe containment measures adopted, Italy has reported and continue to report a high number of infections (on 15th May 2021, 4,126,163 cumulative cases and 122,228 deaths) (7). Among the various NPI, many countries closed schools and kindergartens during lockdowns, resulting in substantial reduction in social interactions. To-date, the effect of school closure is seemingly associated with beneficial effects on overall mortality per week, (8) and has probably different effects depending on the age of children attending schools (9, 10). There is a general consensus on NPI effectiveness in containing SARS-CoV-2, even if before the COVID-19 pandemic evidence supporting isolate NPIs was low (11). Apart from those related to SARS-CoV-2, many studies report a reduction of hospitalization due to lower respiratory tract infections (LRTIs) caused by seasonal respiratory viruses since the first lockdown in March 2020 (12–21). This trend is confirmed by weekly reports of epidemic Influenza virus infection, which show a striking reduction of cases of influenza when compared to previous years (22). Since youngest children are not required to wear personal protective equipment in many Countries, and management of personal hygiene is often not easy, a reduction of respiratory infections may have an environmental explanation.

To our knowledge no study to date compared clinical and microbiologic features of LRTIs, a leading cause of morbidity and mortality in pediatric patients (12, 23, 24) in younger children before and after the documented spread of SARS-CoV-2.

## Materials and Methods

### Design, Setting, and Patients

We performed a retrospective observational study at a tertiary care Hospital (IRCCS Fondazione Cà Granda Ospedale Maggiore Policlinico) of Milan, Northern Italy, in three consecutive winter seasons (2018/2019, 2019/2020 and 2020/2021) from the 1st of November to the last day of February. All hospitalized children under 2 years old diagnosed with LRTI, intended as bronchiolitis or community acquired pneumonia, who underwent a nasopharyngeal aspirate (NPA) for detection of viruses were included. Diagnosis of LRTI was clinically suspected at the admission and confirmed by an expert physician as discharge diagnosis. The NPA for viruses detection tested nucleic acid of respiratory viruses by a multiplex polymerase chain reaction (Allplex Respiratory panel Kit) targeting Influenza virus A and B, Respiratory Syncytial Virus (RSV) (strains A and B were not detectable by this method), Metapneumovirus, Human Bocavirus, Rhinovirus, Coronavirus (subtypes OC43, 229E, and NL23 were not distinguishable by this method), Parainfluenzavirus (strains 1, 2, 3, and 4 were categorized by this method), Adenovirus, Enterovirus (this method can't differentiate Coxsackie from Echoviruses and Polioviruses). Cytomegalovirus was tested only when clinically suspected. In the 2020/2021 season two NPAs for detection of SARS-CoV-2 were performed for each patient (Allplex SARS-CoV-2 Kit). We retrospectively collected data on sex, age in months, selected common risk factors for respiratory infections, treatment and hospitalization days. The institutional ethics board of IRCCS Fondazione Ca' Granda Ospedale Maggiore Policlinico, Milan, approved this study which included a waiver of informed consent because of the retrospective nature of the investigation.

### Statistical Analysis

The proportion of positive NPAs from 1st November to the last day of February were calculated for each winter season in the study (2018/2019; 2019/2020; 2020/2021). The incidence of hospitalization for LRTIs for single viruses was estimated by the mean numbers of events in 2018/2019 and 2019/2020 winter seasons, called pre-COVID-19 seasons. This average was compared with the number of events in 2020/2021 winter season (COVID-19 season group) using the univariate lower tail test of Poisson distribution. Data from pre-COVID-19 seasons and 2020/2021 season were compared. Descriptive statistics were performed: continuous data are presented as median and interquartile range, and categorical data as numbers and percentages. The χ2 test or Fisher's exact test were used for categorical variables, the Mann-Whitney *U*-test for continuous ones. Statistical significance was considered as a *p*-value under 0.05. Statistical analysis was performed using R software (version 3.6.0 for Windows).

## Results

During the observational period, 183 children under 2 years of age with a diagnosis of LRTI were hospitalized. Five patients were excluded, three because they did not undergo a NPA for virus detection, two because of missing data. Four of the excluded patients were admitted during the 2019/2020 winter season, the other one during the 2020/2021 winter season. Thus, a total of 178 patients were enrolled in our study: 66 patients were enrolled in winter season 2018/2019 (54 from the Pediatric Unit and 12 from the Pediatric Intensive Care Unit, respectively), 96 were enrolled in winter season 2019/2020 (78 from the Pediatric Unit and 18 from the Pediatric Intensive Care Unit, respectively), 16 patients were enrolled during winter season 2020/2021, all from the Pediatric Unit. Compared with the average number of hospitalized children in the pre-COVID-19 seasons (81 cases), during 2020/2021 the reduction was 80%. The reduction was statistically significant (cumulative Poisson probability, *p* < 0.00001).

### Demographics, Risk Factors and Comorbidities

Demographic characteristics and risk factors are summarized in Table 1. Our cohort included 94 males (52,8%) and 84 females (47,2%), with a median age of 5 months (IQR 2–13). Thirty-seven patients (20,8%) had a diagnosis of one or more concurring chronic diseases, cardiovascular diseases (14), gastrointestinal disease (9), neurologic disease (9), pulmonary disease (8), and hepatic disease (2), respectively; 16 had multi-organ disease. As concerns other risk factors traditionally linked with LRTIs in infants, 43 patients (24,1%) were born prematurely (26, > 34 weeks gestational age, GA, and 17, < 34 weeks GE), 65 patients (36,5%) were exclusively fed with formula milk from birth, 42 patients (23,6%) were exposed to second-hand smoke. There were some differences between the two groups: hospitalized children during 2020/2021 were older (median age 11.8 months IQR 3–16 vs. 4.3 months IQR 2–10 months; *p* = 0.04) and they had more chronic diseases (44 vs. 18%, *p* = 0.02).

**Table 1:** Viral detections, demographic characteristic and risk factors in pre-COVID-19 seasons and 2020/2021.

	**Pre-COVID-19 seasons** **(n = 162)**		**COVID-19 season** **(n = 16)**		
	**Number of measurements**	***n* (%)**	**Number of measurements**	***n* (%)**	***P-value***
**Demographics**					
Female	162	79 (49)	16	5 (31)	0.18
Male	162	83 (51)	16	11 (69)	0.18
Age in months, median (IQR)	162	4.3 (2–10)	16	11.8 (3–16)	0.04
**Risk factors**					
Comorbidity	162	30 (18)	16	7 (44)	0.01
Prematurity	162	39 (24)	16	4 (25)	1
Exclusively formula milk	152	63 (41)	9	2 (22)	0.31
Passive smoking	137	42 (31)	4	0 (0)	0.32
Older siblings	147	117 (80)	16	10 (62)	0.12
Day-care attendante	153	16 (10)	15	3 (20)	0.38
**Nasopharyngeal aspirate**					
Negative	162	5 (3)	16	2 (12.5)	0.12
Positive	162	157 (97)	16	14 (87.5)	0.12
Single-detections	162	102 (63)	16	8 (50)	0.57
Co-detections	162	55 (34)	16	6 (37.5)	0.57
Negative	162	5 (3)	16	2 (12.5)	0.12

### Respiratory Virus Circulation

**Figure 1:**
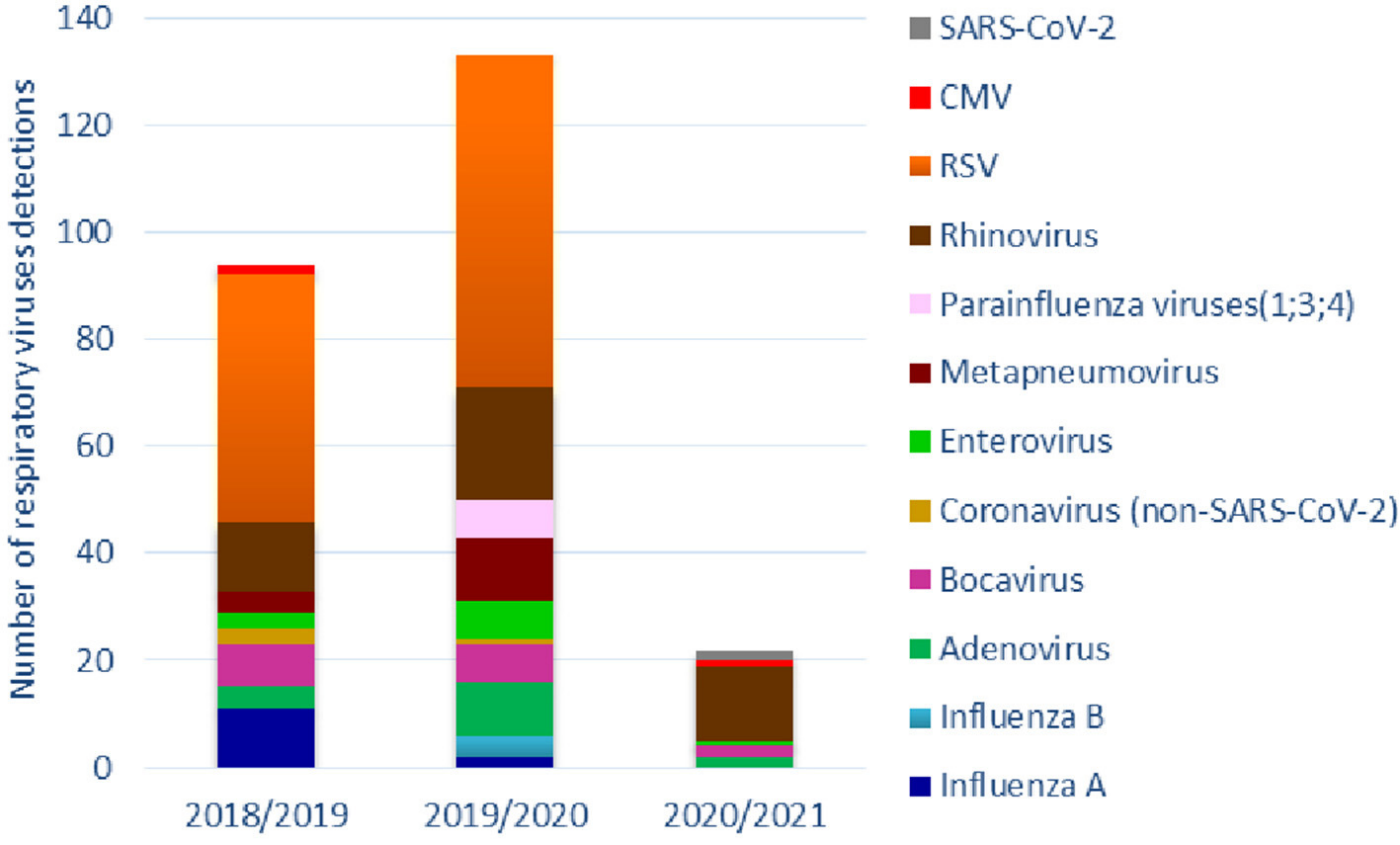
Cumulative respiratory viruses detections by year and viral species.

Overall, 171 patients (96%) had a positive NPA: 110 of them were positive for a single virus, 61 for co-detections, for a total of 249 viruses detected. The proportion of negative, positive and co-detections did not significantly differ between pre-COVID-19 seasons and COVID-19 season (Table 1). The prevalence of single viruses by year is summarized in Table 2. Figure 1 shows the cumulative detection of viruses by year. Table 3 compares the prevalence of single viruses in the COVID-19 season as compared to the average of pre-COVID-19 ones. There was a significant reduction of RSV (0 cases vs. 54 average cases, *p* < 0.00001), influenza A and B (0 cases vs. 6.5 average cases, *p* = 0.0015), Adenovirus (2 cases vs. 7 average cases, *p* = 0.03), Bocavirus (2 cases vs. 7.5 average cases, *p* = 0.02), Enterovirus (1 case vs. 5 average cases, *p* = 0.04), Metapneumovirus (0 cases vs. 8 average cases, *p* = 0.0003), Parainfluenza viruses (0 cases vs. 3.5 average cases, *p* = 0.03) from pre-COVID-19 seasons and 2020/2021 season. No significant difference was found for Rhinoviruses (14 cases vs. 17 average cases, *p* = 0.28), other Coronaviruses (0 cases vs. 2 average cases, *p* = 0.13) and Cytomegalovirus (1 case vs. 1 case, *p* = 0.735). During COVID-19 season two of 16 patients tested positive for SARS-CoV-2: both of them had viral co-infections (one patient had a NPA positive for Bocavirus, the other one for Rhinovirus).

**Table 2:** Viral detections by years.

	**2018/2019 (*n* = 66)**	**2019/2020 (*n* = 96)**	**2020/2021 (*n* = 16)**
	***n* (%)**	***n* (%)**	***n* (%)**
Influenza A	11 (17)	2 (2)	0 (0)
Influenza B	0 (0)	4 (4)	0 (0)
Adenovirus	4 (6)	10 (10)	2 (12)
Bocavirus	8 (12)	7 (7)	2 (12)
Coronavirus (non SARS-CoV-2)	3 (5)	1 (1)	0 (0)
Enterovirus	3 (5)	7 (7)	1 (6)
Metapneumovirus	4 (6)	12 (12)	0 (0)
Parainfluenza viruses (1;3;4)	0 (0)	7 (7)	0 (0)
Rhinovirus	13 (20)	21 (22)	14 (87)
RSV	46 (70)	62 (65)	0 (0)
CMV	2 (3)	0 (0)	1 (6)
SARS-CoV-2	0 (0)	0 (0)	2 (12)

*CMV, cytomegalovirus; RSV, respiratory syncytial virus; SARS-CoV-2, severe acute respiratory syndrome coronavirus 2*.

**Table 3:** Average number of viral detections in pre-COVID-19 seasons and viral detections in 2020/2021 season for viral type.

	**Pre-COVID-19 seasons average**	**COVID-19 season**	**Cumulative probability *p*-value**
Influenza A and B	6.5	0	0.0015
Adenovirus	7	2	0.03
Bocavirus	7.5	2	0.02
Coronavirus (non-SARS-CoV2)	2	0	0.135
Enterovirus	5	1	0.04
Metapneumovirus	8	0	0.0003
Parainfluenzae (1;3;4)	3.5	0	0.03
Rhinovirus	17	14	0.28
RSV	54	0	<0.000001
CMV	1	1	0.735

*CMV, cytomegalovirus; RSV, respiratory syncytial virus*.

*P-value of the cumulative probability using one-tail Poisson distribution*.

### Therapies and Outcomes

In the pre-COVID-19 seasons 83% of the patients needed oxygen therapy compared to 43% in the 2020/2021 season (*p* = 0.05). The time of hospitalization was shorter for children during the COVID-19 season than those in the pre-COVID-19 seasons (median 4 days IQR 4–6 vs. median 6 days IQR 5–8; *p* = 0.006). Therapies and outcome measures are summarized in Table 4.

**Table 4:** Therapies and outcomes in pre-Covid seasons and 2020/2021 season.

	***Pre-covid-19 seasons*** ***(n = 162)***	***Covid-19 season*** ***(n = 16)***	
	***n* (%)**	***n* (%)**	***P value***
Antibiotics use	102 (63)	10 (62)	0.97
Antiviral use	16 (10)	2 (12)	0.67
Oxygen therapy	135 (83)	7 (44)	0.046
CPAP	35 (22)	1 (6)	0.2
HFNC	88 (54)	6 (37)	0.2
Salbutamol aerosol	145 (89)	12 (75)	0.1
Mechanical ventilation	7 (4)	0 (0)	1
Hospitalization days, median (IQR)	6 (5–8)	4 (4–6)	0.006

*CPAP, continuous positive airway pressure; HFNC, high-flow nasal cannula*.

## Discussion

Our study suggests that preventing measures against SARS-CoV-2 had a significant impact on the epidemiology of acute respiratory infections in infancy, with consequences affecting particularly the incidence and the etiology of viral respiratory infections. According to our data, there was a dramatic drop in the number of hospitalizations for LRTIs in children under 2 years old when we compared the 2020/2021 winter season with the two seasons prior to the emergence of SARS-CoV-2 in Italy, with an 80% reduction. The reasons for these findings are likely multifactorial. Although the effectiveness of NPIs in preventing the circulation of respiratory viruses is still widely debated, published evidence suggests that wearing facial masks is effective if combined with other NPIs, such as social distancing and avoidance of overcrowding (25), and that hand hygiene reduces respiratory illnesses (11). Among the most important risk factors for respiratory infections in children, childcare attendance is known to play an important role (26). Hence, the decision of the Italian Government to close day-care centers and schools has limited close contact between children and with teachers, possibly contributing to limit the spread of infections. Moreover, the use of face masks, although not recommended for children younger than 2 years (27), could have reduced infections in household members, lowering the viral shedding in exhaled breath (28). Some authors postulated that the effectiveness of these interventions in reducing viral transmission varies depending on the characteristics of the virus, especially the duration of pre-symptomatic shedding and incubation period (9). If the transmission occurs mainly before the onset of symptoms, NPIs targeting symptomatic patients could have a limited efficacy in reducing the viral spread.

We also report a significant modification in the viral etiology of LRTIs. During the COVID-19 season a total or substantial decline was detected in the prevalence of RSV, Influenza A and B, Metapneumovirus and Parainfluenza viruses, compared to the previous years. We also observed a strong reduction in prevalence of Adenovirus, Bocavirus and Enterovirus, although less impressive than the aforementioned. In contrast, no differences were seen for Rhinovirus, which was the most common virus identified during the COVID-19 season (up to 87% of the positive NPAs). Interestingly, our findings showed that the most common seasonal viruses (e.g., RSV, Influenza, Parainfluenza and Metapneumoviruses) were strongly curtailed in COVID-19 season. Similar results have been described by recent studies worldwide (16, 19, 20, 29). We speculate that seasonal respiratory viruses with an incubation period shorter than SARS-CoV-2 may have been influenced more and earlier by lockdown and distancing measures. Moreover, SARS-CoV-2 could originate interference mechanisms in order to reduce other respiratory viral infections. Yet, studies performed so far are contrasting and not conclusive (18, 21). Indeed, some authors reported a significant rate of viral coinfection with SARS-CoV-2 (30, 31). In our population the proportions of negative, positive and co-detections did not significantly differ between COVID-19 season and the previous ones, but the low rate of SARS-CoV-2 infections (only 2 out of 16 patients) does not allow solid conclusions. The virus structure could play a role too. It is likely that public health measures and NPIs have a stronger impact on enveloped viruses. In fact, the absence of an envelope might explain why Rhinovirus has represented the most frequent virus in our study-population during the last season, and why other respiratory viruses such as Adenovirus and Bocavirus were less affected by the use of face masks and social distancing. Non-enveloped viruses have a greater stability in the environment and are able to survive for extended periods outside the host (32). This makes transmission through droplets less essential for their diffusion, hence their ability to overcome NPIs.

In our study, children hospitalized for LRTIs during the COVID-19 season were older and with more comorbidities, their hospital stay was shorter and oxygen therapy was used less frequently. In addition, no patient during the 2020/2021 season needed admission to the Pediatric Intensive Care Unit, nor mechanical ventilation. These results suggest that infections during the COVID-19 season were milder, affecting predominantly children with an underlying predisposing disease, and that our findings are not influenced by an underutilization of health care (33). A possible explanation may be the change of etiology, in particular the disappearance of RSV, which is known to cause more severe LRTIs (12).

Some limitations of this study should be acknowledged. First, we report a single-center experience, although it is the main Hospital in the center of Milan, the largest city of the Lombardy, that has been the epicenter of the COVID-19 pandemic in Italy. Second, we performed a retrospective analysis, taking into account only children under 2 years of age who required hospitalizations, thus we do not have the whole picture of the prevalence of these viruses in the general population. Data refer to three winter seasons only, thus the estimation of the “typical” pre-COVID season pattern of hospitalization may be subject to a larger random variation.

However, to our knowledge, this is the first study comparing the epidemiology and the role of viruses in LRTIs in children across three winter seasons at the turn of SARS-CoV-2 spread in Italy. Although these preliminary single-center findings need confirmation from a larger surveillance, they are consistent with emerging reports from Brazil (20), Alaska (34), Australia (35), Finland (36), New Zeland (19), France (37), US (38) and Italy (39).

The long-term consequences of the different exposure to viruses in early infancy of children born in 2020 need monitoring at multiple levels, within a range covering the general health of the infant population as well as environmental micro-ecology.

## Data Availability Statement

The raw data supporting the conclusions of this article will be made available by the authors, without undue reservation.

## Ethics Statement

The studies involving human participants were reviewed and approved by the institutional ethics board of IRCCS Fondazione Ca' Granda Ospedale Maggiore Policlinico, Milan. Written informed consent from the participants' legal guardian/next of kin was not required to participate in this study in accordance with the national legislation and the institutional requirements.

## Author Contributions

CA and AL had full access to all of the data in the study and take responsibility for the integrity of the data and the accuracy of the analysis. AL, GI, CA, and PM: concept and design. AL, GI, GU, CA, and PM: drafting of the manuscript, critical revision of the manuscript for important intellectual content, and administrative, technical, or material support. GI, AL, GU, GD, PB, SS, RP, CT, SB, CA, and PM: acquisition, analysis, or interpretation of data. AV: statistical analysis. All authors contributed to the article and approved the submitted version.

## Conflict of Interest

The authors declare that the research was conducted in the absence of any commercial or financial relationships that could be construed as a potential conflict of interest.

## Publisher's Note

All claims expressed in this article are solely those of the authors and do not necessarily represent those of their affiliated organizations, or those of the publisher, the editors and the reviewers. Any product that may be evaluated in this article, or claim that may be made by its manufacturer, is not guaranteed or endorsed by the publisher.
