# Corrigendum: Disappearance of seasonal respiratory viruses in children under two years old during COVID-19 pandemic: A monocentric retrospective study in Milan, Italy

**DOI:** 10.3389/fped.2022.1006948

**Published:** 2022-08-23

**Authors:** Giulio Ippolito, Adriano La Vecchia, Giulia Umbrello, Giada Di Pietro, Patrizia Bono, Stefano Scalia Catenacci, Raffaella Pinzani, Claudia Tagliabue, Samantha Bosis, Carlo Agostoni, Paola Giovanna Marchisio

**Affiliations:** ^1^University of Milan, Milan, Italy; ^2^Fondazione IRCCS Ca' Granda Ospedale Maggiore Policlinico, Laboratory of Virology, Milan, Italy; ^3^Fondazione IRCCS Ca' Granda Ospedale Maggiore Policlinico, Pediatric Intensive Care Unit, Milan, Italy; ^4^Fondazione IRCCS Ca' Granda Ospedale Maggiore Policlinico, Pediatric Highly Intensive Care Unit, Milan, Italy; ^5^Fondazione IRCCS Ca' Granda Ospedale Maggiore Policlinico, Pediatric Intermediate Care Unit, Milan, Italy; ^6^Department of Clinical Sciences and Community Health (DISCCO), University of Milan, Milan, Italy; ^7^Department of Pathophysiology and Transplantation, University of Milan, Milan, Italy

**Keywords:** respiratory viruses, children, COVID-19, epidemiology, public health

In the published article, an author name was incorrectly written as Stefano Scalia. The correct spelling is Stefano Scalia Catenacci.

The authors apologize for this error and state that this does not change the scientific conclusions of the article in any way. The original article has been updated.

## Publisher's note

All claims expressed in this article are solely those of the authors and do not necessarily represent those of their affiliated organizations, or those of the publisher, the editors and the reviewers. Any product that may be evaluated in this article, or claim that may be made by its manufacturer, is not guaranteed or endorsed by the publisher.

